# Wildfires and Drought Reshape Terrestrial Mammal Communities While Aquatic Prey Sustain Jaguars in Brazil's Northern Pantanal

**DOI:** 10.1111/gcb.70589

**Published:** 2025-10-27

**Authors:** Alan Eduardo de Barros

**Affiliations:** ^1^ Departamento de Ecologia, Instituto de Biociências Universidade de São Paulo São Paulo São Paulo Brazil

**Keywords:** aquatic prey, climate change, drought, jaguars, mammals, terrestrial prey, trophic control, wildfires

Climate change and human activities are driving increasingly frequent and intense droughts and wildfires worldwide, degrading habitats, displacing wildlife, intensifying competition, and disrupting food webs (Pivello et al. 2021; Tomas et al. 2021). These disturbances operate across multiple spatiotemporal scales: drought causes long‐term regional reductions in water availability and primary productivity, while wildfires trigger immediate vegetation loss, direct mortality, and persistent ecological changes (Tomas et al. 2021; Flores et al. 2021). Understanding the individual and combined impacts of drought and fire on biodiversity remains challenging, given their frequent co‐occurrence, unpredictability—especially in the case of wildfires—and scarcity of pre‐ and post‐event data. Meeting this challenge requires robust monitoring capable of capturing wildlife dynamics and ecosystem conditions before, during, and after disturbances.

In a newly published study in *Global Change Biology*, Eriksson et al. (2025) seized the rare opportunity created by the 2020 Pantanal wildfires—one of the most severe drought–fire events in the biome's history—to examine how extreme climatic disturbances reshape top predator dynamics and terrestrial mammal communities, while elegantly addressing fundamental predator–prey ecological questions. The Pantanal, the world's largest tropical wetland (≈179,300 km^2^), is a biodiversity hotspot with seasonally flooded plains, grasslands, savannas, forests, and wetlands that support diverse vertebrate communities (Marengo et al. 2021; Eriksson et al. 2022). These resource‐rich landscapes sustain exceptionally high jaguar ( *Panthera onca* ) densities (6.6–12.3 individuals per 100 km^2^), especially in areas with abundant aquatic prey (Eriksson et al. 2022), and harbour the largest individuals recorded across the species' range. Although drought and fire are part of the Pantanal's natural regime, their impacts have been intensified by climate change, deforestation, agricultural expansion, infrastructure development, and hydrological alterations, which together exacerbate drought conditions and increase wildfire risk (Libonati et al. 2020; Marengo et al. 2021).

In 2020, the Pantanal experienced its most severe drought in 50 years (Marengo et al. 2021), accompanied by unprecedented wildfires that burned approximately 30% of the biome (Libonati et al. 2020; Pivello et al. 2021) and killed about 17 million vertebrates (Tomas et al. 2021). Beyond direct mortality, many animals were displaced or likely succumbed to dehydration and starvation, though the extent of these indirect effects remains unknown. The fires overlapped 79% of 48 monitored jaguar home ranges and affected 45% of the biome's estimated jaguar population (De Barros et al. 2022). Recovery from such extreme events depends on disturbance severity, time elapsed, and vegetation resilience. While savannas are relatively fire‐adapted, riparian forests and forest patches may take decades to regenerate (Flores et al. 2021). Post‐fire dynamics are further shaped by spatial variation in terrestrial and aquatic resources, vegetation recovery, and shifts in predator–prey interactions (Eriksson et al. 2022; Doherty et al. 2022). These extreme drought–fire events thus serve as rare ‘natural experiments’, offering unique opportunities to investigate how disturbances reshape ecological communities.

Using a long‐term dataset and a before–after–control–impact (BACI) framework, Eriksson et al. (2025) examined how the 2020 wildfires and drought affected jaguars and other mammals in Brazil's northern Pantanal, also testing how aquatic resources influence the terrestrial community. They combined camera trap data and jaguar scat analyses collected before, during, and after the fires to address four main objectives: (i) assess immediate and short‐term impacts of fire on jaguar demography, abundance, activity, space use, and diet; (ii) evaluate changes in mammal species richness and relative abundance; (iii) determine whether fire, drought, or both were the main drivers of community change and (iv) test whether the terrestrial community is regulated by top‐down predation—potentially through apparent competition, where aquatic prey sustain high jaguar densities and intensify predation on terrestrial mammals—or by bottom‐up resource availability, indicated by increases in terrestrial prey following habitat recovery. By integrating 3 years of pre‐fire data, observations during and immediately after the fires, and 2 years of post‐fire monitoring across impacted and control sites, the study provides rare and timely evidence disentangling the effects of wildfire from those of prolonged drought.

Eriksson et al. (2025) detailed how and when wildfires and drought affect jaguars and other terrestrial mammals in the Pantanal, influencing activity, abundance, demography, space use, diet, and broader ecological dynamics. Their study also highlights the uniqueness of the Taiamã Ecological Station and its surroundings, emphasizing its role as both a climate refuge and a major source of aquatic food that shapes predator–prey interactions. Jaguar activity declined immediately after the fire (*β* = −1.78, *p* < 0.001), reflecting a short‐term response to habitat disturbance, but subsequently rebounded. Over the longer term, jaguars showed no consistent preference for burned versus unburned areas; however, activity decreased in a previously important hotspot, likely due to high burn severity (see figure 5 and figure S1 in Eriksson et al. 2025). Despite these disturbances, most resident jaguars maintained home ranges, with recapture rates of 45%–48% pre‐fire and 43%–70% post‐fire. When combined with immigration from surrounding areas, the estimated number of adults nearly doubled (from 21–29 to 49–50). One year post‐fire, jaguar abundance and recruitment increased markedly, with cub numbers rising fourfold (from 3 to 13–14), even under severe drought conditions.

Seasonal flooding contracted by 62% across the broader Pantanal but only 19.7% within Taiamã, underscoring its role as a refuge and suggesting a trend toward an inverse relationship between jaguar abundance and dry‐season river levels (*R* ^2^ = 0.51, *p* = 0.07). Similarly, mammal species richness and relative abundance were more strongly tied to drought‐driven changes in water availability than to fire, with richness showing a strong negative correlation with water level (*R* ^2^ = 0.86, *p* = 0.004). Despite greater terrestrial prey availability, jaguars continued to specialize in aquatic prey (81%–83% of diet before and after fire), supporting the hypothesis that aquatic resources buffer predation on terrestrial mammals.

These findings underscore the importance of understanding how drought and wildfires affect wildlife in the Pantanal, with valuable implications for jaguar conservation and broader ecological dynamics. Yet the unique conditions of Taiamã—its relative drought resistance, rapid post‐fire recovery, and abundant aquatic prey—limit generalization across the biome. Elsewhere, harsher droughts, slower vegetation recovery, stronger reliance on terrestrial prey, and the absence of refugia may intensify the consequences of climate‐driven disturbances. Methodological limitations also warrant caution: camera‐trap sampling was restricted to gallery forests near water sources, which, while critical jaguar habitat, does not capture use of other habitats. Broader, systematic or stratified sampling encompassing all major habitats used by jaguars would strengthen future assessments. Such caveats are not unique to the Pantanal and may be even more pronounced in other biomes and ecoregions.

Short‐term behavioural responses to fire remain largely unexplored. How jaguars and other species adapt to different burn frequencies, magnitudes, and extents—or after rescue and re‐release—remains unknown. Previous work shows that other human disturbances reduce jaguar movement, activity, and foraging, with tolerance declining as disturbance magnitude increases (Morant et al. 2025). However, no habitat selection studies have directly examined the effects of the increasing severity of fire and drought regimes. Future research could integrate GPS telemetry and accelerometers to assess fine‐scale movement, space use, energy expenditure, and behaviour before, during, and after disturbances. Movement data collected at short intervals and analysed within a BACI framework would complement camera‐trap monitoring by comparing collared individuals in burned and unburned areas. Opportunistic data from rescued and re‐released jaguars could further reveal short‐term behavioural adjustments.

Long‐term datasets spanning multiple generations are essential for drawing robust conclusions about population densities and ecological dynamics, as well as validating patterns inferred from relative abundance and occurrence data. Continued monitoring is crucial to distinguish short‐term spatial shifts from genuine demographic recovery after extreme climatic events. Multi‐site monitoring under contrasting fire conditions over time would further strengthen assessments of space use, survival, and movement shifts, providing critical insights for conservation planning. Future research could also explore whether resource availability and maternal teaching influence prey selection. Such efforts will advance understanding of jaguar ecology, clarify ecological processes in regions with limited water or scarce aquatic prey, and improve predictions of tropical wetland resilience under intensifying climate stress.

From a broader perspective, Eriksson et al. (2025) demonstrate the value of integrating long‐term datasets with opportunistic monitoring during extreme events—an approach applicable across ecosystems facing increasing climate volatility. Their use of BACI designs, camera‐trapping, and dietary analyses provides a robust framework for disentangling disturbance effects, which could be further enhanced through complementary methods such as telemetry, accelerometry and demographic monitoring. Embedding such approaches within coordinated, cross‐site networks would strengthen our capacity to anticipate and compare biodiversity responses to fire and drought globally. Notably, these advances can also reinforce mitigatory strategies by grounding fire management, deforestation control, and forest restoration in evidence of species and ecosystem responses.

In conclusion, Eriksson et al. (2025) provide key ecological insights for biodiversity conservation as well as wildlife and landscape management amid escalating climate‐driven disturbances. As extreme droughts and wildfires intensify, understanding their temporal effects and species' ecological responses is essential. Equally important are efforts to protect climate refugia, sustain long‐term monitoring, and apply emerging ecological knowledge to guide conservation strategies that anticipate and reduce future impacts.

## Conflicts of Interest

The author declares no conflicts of interest.

## Linked Articles

This article is a Invited Commentary on Eriksson et al., https://doi.org/10.1111/gcb.70344.

## Data Availability

The author has nothing to report.
